# French Quinine

**Published:** 1873-12

**Authors:** 


French Quinine.—At the Philadelphia College of Pharmacy, Mr. Gaillard spoke of having received a sample of what purported to be French quinine, from a friend in the South, who had been offered the article at a very low price, and had sent him a portion to ascertain why it could be sold so low. It proved to be the old fraud—muriate of cinchonia.

Prof. Maisch read an extract from the "Circular of the Philadelphia Drug Exchange," in relation to this subject, as follows:

*Cinchonia Muriate.* From the *Druggists' Circular*, New York, October, 1873, we extract the following statement, reported in the

Transactions of the American Pharmaceutical Association at Richmond:

"Prof. Maisch drew attention to the fact that very large quantities of muriate of cinchonia had been put up in the style of French quinine, and having an imitation of Pelletier's label upon it, and that it had been extensively introduced in the Southern States.

"Dr. Squibb said that some of the manufacturers of quinia were, in part, responsible for this attempt to defraud the people, as they, in the course of their manufacture, accumulated large quantities of the cinchonia salts, and they disposed of them indiscriminately to any who applied for them."

We take occasion to say that, so far as *American* manufacturers of sulph. quinia are concerned, (1) they do not dispose of the cinchonia salts indiscriminately to any who apply for them, but only to regular customers who pay for them ; and, (2) so far from being responsible for this attempt to defraud the people, they purposely avoid handling *muriate of cinchonia*—they do not make the article.

We consider that this statement is eminently due to our friends who make sulph. quinia here, for they have not only declined making, but refuse to deal in, the article of *muriate of cinchonia*, on account of its close resemblance to *sulphate of quinia*.

*Muriate of cinchonia* is largely sold in *Europe*, but not in this country, so far as sulph. quinia manufacturers are interested.

